# Cerebroprotective Effects of 2-Ethyl-6-methyl-3-hydroxypyridine-2,6-dichlorophenyl(amino)phenylethanoic Acid in the Treatment of Purulent Meningitis

**DOI:** 10.3390/biomedicines9030285

**Published:** 2021-03-11

**Authors:** Alina Agarkova, Mikhail Pokrovskii, Pavel Kolesnichenko, Vladimir Gureev, Oleg Gudyrev, Anna Peresypkina, Vladislav Soldatov, Arkadii Nesterov, Tatyana Denisyuk, Mikhail Korokin

**Affiliations:** 1 Department of Pharmacology and Clinical Pharmacology, Belgorod State National Research University, 308015 Belgorod, Russia; pokrovskii@bsu.edu.ru (M.P.); kolesnichenko_p@bsu.edu.ru (P.K.); produmen@yandex.ru (V.G.); gudyrev@mail.ru (O.G.); peresypkina_a@bsu.edu.ru (A.P.); soldatov_v@bsu.edu.ru (V.S.); korokin@bsu.edu.ru (M.K.); 2 Department of Pathology, Belgorod State National Research University, 308015 Belgorod, Russia; n-a-vit@yandex.ru; 3 Department of Pharmacology, Kursk State Medical University, 305000 Kursk, Russia; denitatyana@yandex.ru

**Keywords:** pneumococcal meningitis, bacterial meningitis, treatment of bacterial meningitis, cerebroprotection

## Abstract

Purulent meningitis (PM) is a severe disease, characterized by high mortality and a formation of a residual neurological deficit. Loss of treatment of PM leads to the lethal outcome in 100% of cases. In addition, death and the development of residual neurological complications are possible despite adequate therapy. The aim of the study was to evaluate the cerebroprotective effects of a new pharmacological compound 2-ethyl-6-methyl-3-hydroxypyridine-2,6-dichlorophenyl(amino)phenylethanoic acid (EMHDPA) on the bacterial purulent meningitis in a model of experimental pneumococcal meningitis. Meningitis was simulated by intrathecal injection of the suspension containing *Streptococcus pneumoniae* at the concentration of 5 × 10^9^ CFU/mL. The cerebroprotective effect was evaluated by survival rates, the severity of neurological deficit, investigatory behaviors, and results of short-term and long-term memory tests. The group administered with EMHDPA showed high survival rates, 80%. Animals treated with the studied compound showed a higher clinical assessment of the rat health status and specific force, and a lesser intensity of neurological deficit compared to the control group (*p* < 0.05). Locomotor activity of the animals treated with EMHDPA was significantly higher compared to the control group (*p* < 0.05). There is a decrease in the activity of all estimated indicators of oxidative stress in the group administered with 2-ethyl-6-methyl-3-hydroxypyridine-2,6-dichlorophenyl(amino)phenylethanoic acid relative to the control group: a decrease in the activity of catalase—17%, superoxide dismutase—34%, malondialdehyde and acetylhydroperoxides—50%, and nitric oxide—85% (*p* < 0.05). Analysis of the data obtained during the experiment leads to the conclusion about the effectiveness of 2-ethyl-6-methyl-3-hydroxypyridine-2,6-dichlorophenyl(amino)phenylethanoic acid in the treatment of the experimental PM.

## 1. Introduction

Purulent meningitis (PM) is usually a severe disease characterized by high mortality and the formation of a residual neurological deficit after the disease. Loss of treatment of PM leads to the lethal outcome in 100% of cases. In addition, death and the development of residual neurological complications are possible despite adequate therapy.

The incidence of bacterial meningitis is about five cases per 100,000 adults per year in developed countries and can be 10 times higher in less developed countries. The predominant pathogens in adults are *Streptococcus pneumoniae* (pneumococcus) and *Neisseria meningitidis* (meningococcus), which account for 80% of all cases of bacterial purulent meningitis. Pneumococcal meningitis is characterized by the highest mortality and severity of the course among all BGM [1].

Due to the vaccination programs against meningococcal, hemophilic, and pneumococcal infections, the morbidity of PM has decreased worldwide [2]. The second place among the pathogens of PM is *S. pneumoniae*, second only to meningococcus [3,4,5,6,7].

Children from 0 to 4 years of age are most often affected by PM, and the morbidity rate in this age group is 10 per 100,000 children. In adults, the morbidity of PM is significantly less, 1–2.5 per 100,000 per year. Among adults, the maximum morbidity is observed at the age of 45–64 [4,5,8].

The mortality rate for PM ranges from 28% to 60% in different countries [3].

The outcome of PM depends on the properties of the pathogen, its sensitivity to antibacterial drugs, as well as on the age and premorbid background of the patient.

PM can be both primary and secondary. Primary meningitis develops against a background of complete well-being, without a previous disease. Secondary meningitis occurs already against the background of an existing infectious pathology. The portal of entry of infection in primary meningitis is the mucous membranes of the oropharynx and bronchi. In secondary meningitis, the pathogen penetrates the blood–brain barrier (BBB) from the existing site of infection in the body (otitis, pneumonia).

The severity of the meningitis is assessed by the severity of meningeal and cerebral syndromes, the level of pleocytosis in the cerebrospinal fluid, and the presence or absence of complications.

PM is characterized by a peracute clinical pattern with a rapid increase in cerebral and meningeal syndrome. In PM, meningeal syndrome is prominent from the first day of the disease. Early focal symptoms are associated with the development of meningoencephalitis. Clinically, this is manifested by convulsive syndrome and loss of consciousness. Due to the development of a purulent-adhesive process in the pia mater and panvasculitis, the recovery of the cerebrospinal fluid in PM lasts from two to four weeks. With PM, relapses are possible, most often occurring in violation of the dura mater integrity. If the PM clinical pattern has a long-term comatose condition and a long-term convulsive syndrome (more than 12 h), the prognosis is usually unfavorable. The immediate cause of death is a brainstem herniation, which develops as a result of brain edema. Neurological deficit develops in 50% of PM cases. Sensorineural hearing loss, speech and motor disorders, and symptomatic epilepsy are the most common [9].

Combination therapy of the bacterial pneumococcal meningitis includes etiotropic antibacterial therapy, adjuvant hormone therapy, symptomatic therapy, and neuroprotective therapy.

Antibacterial therapy should be started no later than 1 h after the diagnosis of PM. Antibiotics are administered only parenterally. As a rule, antimicrobial therapy is prescribed empirically in 75–90%. In accordance with the recommendations of the European Society of Clinical Microbiology and Infectious Disease 2016, the choice of empirical antibacterial therapy depends on the patient’s age and the sensitivity of pneumococcus to penicillin and third-generation cephalosporins in this region [4,10]. If the pneumococcal etiology of meningitis is suspected, ceftriaxone is most often the frontline treatment, provided that the isolated strain is sensitive to penicillin. The correction of antibacterial therapy is carried out in accordance with the sensitivity of the isolated microorganism, based on the results of liquor culture. When PM is caused by a third-generation cephalosporin-resistant strain of pneumococcus, vancomycin or rifampicin should be added to the third-generation cephalosporins. A combination of vancomycin + rifampicin can also be used [4]. Alternative antibacterial drugs in the treatment of PM are cefepime and carbapenems. The recommended duration of the treatment is 10–14 days [4,11]. The criteria for evaluating the effectiveness of the treatment are normalization of body temperature, the patient’s clinical condition (no complaints, meningeal symptoms), and the absence of cerebrospinal fluid pleocytosis.

As it is known, the result of effective antibacterial therapy is the death of pneumococci, which is accompanied by the release of cellular debris. The more of these substances, the more pronounced the inflammatory response and the more massive area of damage to neurons. These observations formed the basis for the search for non-bacteriolytic antimicrobials [12,13]. Currently, studies on rifampin, moxifloxacin, and daptomycin are being conducted [4,14].

The effectiveness of adjuvant hormone therapy with dexamethasone in PM has been proven to improve survival rates [15]. Dexamethasone should be administered before the first antibiotic injection [4]. This procedure reduces the severity of inflammation in the subarachnoid space in PM and, accordingly, the area of damage to neurons [16,17]. However, it should also be kept in mind that as a result of treatment with glucocorticosteroids, the permeability of the blood–brain barrier to antibiotics decreases. This is especially important in the treatment of PM with vancomycin [18].

Depending on the clinical manifestations, osmotic therapy with diuretics, antipyretic therapy, anticonvulsants, immunotherapy, and anticoagulants are used individually for PM. Neuroprotective therapy is widely used to improve the outcome of the disease and reduce the severity of residual neurological deficit. In clinical practice, nootropic drugs, GABA receptor agonists with neuroprotective, neurometabolic, neurotrophic, sedative, and anticonvulsant effects are actively used. Their benefit in PM is obvious. However, there have been no studies that investigate these drugs in PM in clinical trials.

3-hydroxypyridine derivatives have long been used in medicine and pharmacology. They have a variety of pharmacological effects—neuroprotective, antihypoxic, nootropic, anti-ischemic, anti-stress, vegetotropic, cardioprotective, geroprotective, anxiolytic, anticonvulsant, anti-alcohol. The pharmacological effect of the compounds of this group is mainly aimed at blocking the processes of lipid peroxidation and activation of antioxidant defense enzymes (for example, superoxide dismutase), which are involved in the formation and consumption of reactive oxygen species and lipid peroxides. With the development of damage in the brain tissue, there is, first of all, a violation of the integrity of the cell membrane (namely, an increase in the viscosity of its bilipid layer, depolarization, a change in the sensitivity thresholds of neurons) [19,20]. Therefore, we decided to investigate a new derivative of 3-hydroxypyridine as a cerebroprotector in the treatment of BGM.

Objective: to study the cerebroprotective effects of a new pharmacological compound 2-ethyl-6-methyl-3-hydroxypyridine-2,6-dichlorophenyl(amino)phenylethanoic acid (EMHDPA) in the treatment of bacterial purulent meningitis, on the example of the pneumococcal, in experiment.

## 2. Experimental Section

### 2.1. Animals

The experiments were approved by the Belgorod State National Research University, Local Ethics Committee, Belgorod (Protocol #12/18 from 11 September 2018. The study was performed in 38 mature female Wistar rats weighting 230–260 g. Ethical principles of handling laboratory animals were observed in accordance with the European Convention for the Protection of Vertebrate Animals Used for Experimental and Other Scientific Purposes, CETS No. 123. The animals were housed in an animal facility with a 12 h day/12 h night cycle and provided with a standard laboratory diet and water. During the period of the study, the animals were healthy, with no changes in behavior, appetite, or sleep–wake schedule. For 18 h before the experiments, the animals were under the condition of complete food deprivation with free access to water.

### 2.2. Preparation of Microbial Culture

*S. pneumoniae* serotype 3 was used as the pathogen. The microorganism was cultured in 10 mL of Todd Hewitt broth for 12 h, then diluted with fresh broth and grown to the logarithmic phase. The finished culture was centrifuged for 10 min at a speed of 5000 rpm. Then suspending had been performed in a sterile saline solution until a concentration of 5 × 10^9^ CFU/mL was reached [18].

### 2.3. Experimental Model of Meningitis

Pneumococcal meningitis was simulated by the following way. First, using a hair removal cream, the hair was removed in the area of the intended puncture. Then antiseptic preparation was performed. The animal was placed in prone position and its head was tilted down at an approximately 45-degree angle, so that a rhomboid fossa was visualized, between the occipital protuberance and the atlas. The subarachnoid space was punctured using a 23G needle, holding the rat by the pelvic girdle with one hand. Subarachnoid puncture was performed under anesthesia with chloral hydrate 160 mg/kg and Zoletil 60 mg/kg intraperitoneally. To induce meningitis, 10 µL of the suspension containing the *S. pneumoniae* serotype 3 was administered at the concentration of 5 × 10^9^ CFU/mL. Then the animals were returned to their cages. Eighteen hours later, the development of meningitis was confirmed by a quantitative culture of 5 µL of CSF, obtained by the subarachnoid puncture.

### 2.4. Experimental Design

Experimental animals were divided into 3 groups:

(1) intact—uninfected animals (*n* = 10),
(2) control group with simulated pneumococcal meningitis administered with Ceftriaxone only (*n* = 14),
(3) group with simulated pneumococcal meningitis administered with Ceftriaxone and compound 2-ethyl-6-methyl-3-hydroxypyridine-2,6-dichlorophenyl(amino)phenylethanoic acid (*n* = 14).

Treatment was started 18 h after. The animals were treated with Ceftriaxone (100 mg/kg body weight) intramuscularly for 7 days. Ten days later the animals were free of the infection. The absence of the infection was confirmed by the subarachnoid puncture at day 10 and a subsequent negative result of CSF culture [21,22]. EMHDPA was administered intramuscularly 7 h after meningitis induction at the above dosages.

Four animals in groups 2 and 3 were sacrificed 24 h after meningitis induction to assess the parameters of oxidative stress in the brain homogenate, the other half of animals were kept watch over for 10 days, assessing the degree of neurological deficit and behavioral status. Rat brain homogenate was prepared as follows: the animal was decapitated, the brain was extracted and placed in a 0.9% NaCl solution cooled to 0 ± 5 C, then homogenized for 4 min while cooling. Tissue homogenate was prepared at the rate of 100 mg of tissue per 2 mL of saline solution.

The cerebroprotective effect was judged by indicators of mortality, severity of neurological deficit, behavioral status, and the level of oxidative stress indicators (catalase activity, superoxide dismutase, acetylhydroperoxides, nitric oxide metabolites, and malondialdehyde).

### 2.5. Methods for Assessing the Severity of Neurological Disorders

Clinical assessment of the health status of rats at the 1st, 3rd, 5th, 7th, and 8th days after pathology simulation was performed as follows. The rats were weighed, and the severity of the disease was evaluated clinically using the following scale: 1 = coma; 2 = does not turn vertically in a supine position; 3 = turns vertically for 30 s; 4 = minimal ambulatory activity, turns vertically for <5 s; and 5 = normal.

The severity of neurological deficit was evaluated using the neurological deficit assessment scale for meningitis and meningoencephalitis at the 1st, 5th, and 8th day after pathology simulation (Table 1).

The severity of the neurological deficit was assessed daily by the total score. Twenty-one points indicate the absence of neurological deficit, 0 points indicate its maximum severity. The severity of the neurological deficit was assessed as follows: 18 points or fewer—mild, 14–17 points—medium, 13 points or more—heavy.

Using a device for evaluating the muscle strength of the limbs of small laboratory animals, the specific force of rats was determined in the experiment at the 1st, 5th, and 10th day.

Evaluation of the investigatory behaviors of the rats was performed using the Infrared Actimeter (IR Actimeter, Panlab Harvard Apparaturs LE 8825, Barcelona, Spain) at the 1st, 3rd, and 10th days. Motor stereotypy, total activity, maximum speed, rest time, and total distance were evaluated using ActiTrack software (Panlab Harvard Apparaturs LE 8825, Barcelona, Spain).

### 2.6. Method for Assessing Cognitive Impairment

On the 10th day after meningitis induction, the cognitive capacity of rats was evaluated using the object recognition task. This test allows you to evaluate spatial memory. The experiment was performed in an open field made of wood (field size, 40 × 50 cm, wall height, 50 cm). First, a 5 min habituation session was performed, during which the animals freely explored the open field. At this time, there were no objects in the open field. After the habituation session, a training session was conducted: the rats, one by one, examined for 5 min in an open field, in which there were 2 identical objects (objects A1 and A2, both cubes). The objects were located at a distance of 10 cm from the walls in 2 adjacent corners.

Short-term memory (STM) analysis of object recognition was performed 90 min after the training session. The animals were tested for 5 min in the open field, which contained one familiar object (A) and one new object (B, a pyramid with a square base). The recognition index was calculated using the formula TB/(TA + TB) where TA is the time spent studying a familiar object A and TB is the time spent studying a new object B.

Testing of the rats for the analysis of long-term memory (LTM) of object recognition was performed 24 h after the training session. The animals were tested in the open field for 5 min in the presence of one familiar object A and one new object C (a ball with a square base). Recognition memory was evaluated in the same way as in short-term memory analysis. Sniffing (examining the object from a distance of 3–5 cm) or touching the object with the nose and/or forelegs were considered an object study. All the objects used in the test had a similar texture (smooth), color (blue), and size (weight 150–200 g), but different shapes [19].

### 2.7. Method for Assessing Indicators of Oxidative Stress

It is known that many pathological processes in tissues are associated with the formation of reactive oxygen species with high reactivity. In a number of pathological conditions, there is an overconcentration of lipid peroxidation products (LPP). The key enzyme of antioxidant protection is superoxide dismutase (SOD), which catalyzes the enzymatic dismutation of the superoxide-anion radical.

The SOD activity was determined by the spectrophotometric method using the spectrophotometer APEL 330 PD (Tokyo, Japan), based on determining the degree of inhibition of the quercetine auto-oxidation reaction. The amount of the enzyme needed to reduce the rate of quercetine oxidation by 50% was taken as a relative unit of SOD activity.

One of the enzymes of the system of reactive oxygen species inactivation is catalase, which catalyzes the cleavage reaction of hydrogen peroxide. The biological role of the enzyme is to prevent the accumulation of hydrogen peroxide produced by the dismutation of the superoxide anion. Catalase activity was determined by a method based on the ability of hydrogen peroxide to form a stable colored complex with molybdenum salts. The color intensity was measured photometrically at a wavelength of 410 nm using the spectrophotometer Apel 330PD spectrophotometer (Japan). Catalase activity was expressed in mcat/mL.

Malondialdehyde (MDA) is the final product of lipid peroxidation. The determination was performed spectrophotometrically using the spectrophotometer Apel 330 PD (Japan) after extraction with butanol using “TBK-Agat”. The results were expressed in µmol/L.

Acetylhydroperoxides (AHP) are an intermediate metabolite of lipid peroxidation. The determination was made using a mixture of heptane and isopropane with the addition of hydrochloric acid. The resulting heptane layer was measured spectrophotometrically at a wavelength of 233 nm against the control sample. The results were presented in relative units.

Determination of the stable metabolites of nitric oxide was performed by adding Griess reagent to the bioassay. The concentration was measured spectrophotometrically at a wavelength of 540 nm after 5 min of incubation at room temperature.

### 2.8. Histological Study

For histological examination, the brain, liver, kidneys, lungs, heart, spleen, and pancreas were collected and fixed in a 10% solution of buffered neutral formalin (BioVitrum, Russia) for 24–48 h. After that, 2 pieces of 0.2–0.3 cm thick and about 1 cm^2^ in area were cut from each brain sample and 1 piece was cut from the internal organs. The specimens were subjected to standard alcohol processing using the device Leica TP 1020, after which the material was embedded in paraffin Histomix (BioVitrum, Saint-Petersburg, Russia). Histological sections 4–5 microns thick were prepared and stained with hematoxylin and eosin, using the standard protocols, techniques, and devices Leica EG 1150 H, Leica RM 2245, and Leica autostainer XL. To describe the morphological changes in individual brain structures in detail, Nissl staining was used. Immunohistochemical determination of apoptosis was performed with antibodies to the p53 protein and bcl-2, the glial reaction was detected using glial fibrillar acid protein GFAP. Microscopic examination of microsections was performed using a Nikon Eclipse Ni microscope and Nis-Elements BR 4.60.00 software (version 4, Nikon Corporation, Tokyo, Japan).

### 2.9. Statistical Analysis

For all data, descriptive statistics were used, and the data were checked for normal distribution. The distribution type was determined by using the Shapiro–Wilk test. In the case of the normal distribution, the average value (M) and standard deviation (SD) were calculated. In cases of the abnormal distribution, the median (Me) and the quartile range (QR) were calculated. In the normal distribution, the intergroup comparison was performed using one-way ANOVA and post-hoc analysis according to Tukey. In other cases, the intergroup comparison was performed using Kruskal–Wallis and Dunn’s post-hoc tests. Statistical analyses were performed using the R programming language.

## 3. Results

**Figure 1:**
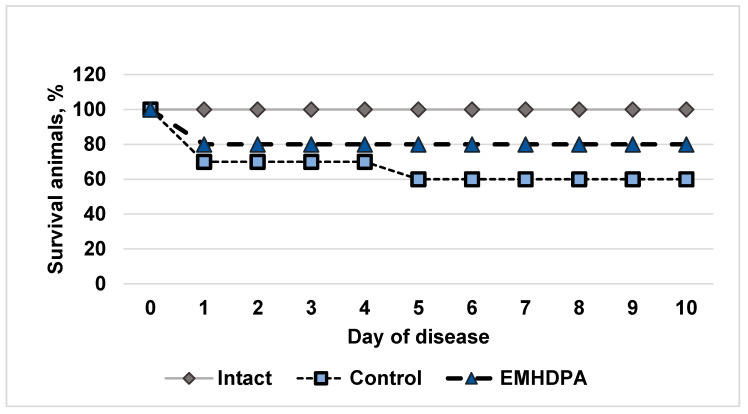
Effect of the studied drugs on rat survival in simulated pneumococcal meningitis in the experiment, %. 1—intact group; 2—control group; 3—group administered with EMHDPA 25 mg/kg.

In the group of intact animals, there was no lethality; in the control group, the survival rate on the 10th day after simulated meningitis was 60% (Figure 1). In the group administered with EMHDPA, there were high survival rates, 80%.

**Figure 2:**
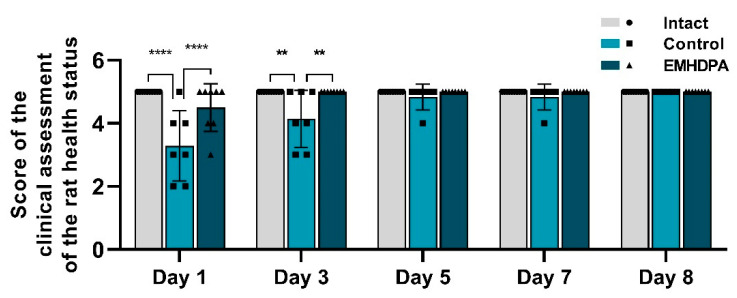
Dynamics of the clinical assessment of health status in the studied groups (by the average score in the group). Note: **—*p* < 0.01; ****—*p* < 0.0001; 1—intact group; 2—control group; 3—group administered with EMHDPA 25 mg/kg.

On the first and third days after meningitis simulation, rats treated with EMHDPA had a statistically significant higher clinical score compared to the control group by 36.7% and 20.8%, respectively (*p* < 0.05) (Figure 2). Recovery of the clinical activity of rats (5 points) in the group treated with EMHDPA, was the fastest on the third day. In the control group, the clinical score rises to the baseline on day 8.

**Figure 3:**
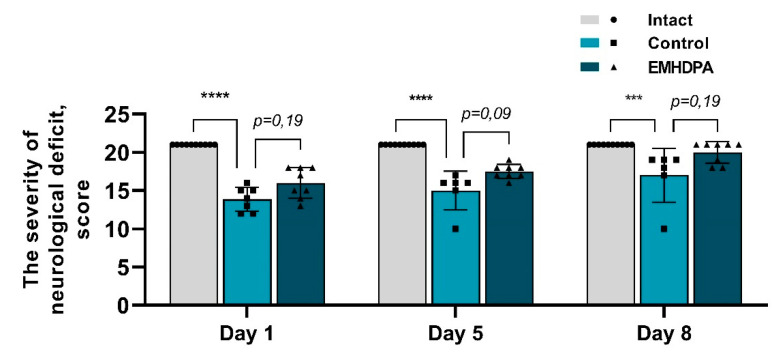
Dynamics of the severity of neurological injuries in the studied groups on the scale of assessment of the severity of neurological deficit in meningitis, meningoencephalitis (by the average score in the group). Note: ***—*p* < 0.001; ****—*p* < 0.0001; 1—intact group; 2—control group; 3—group administered with EMHDPA 25 mg/kg.

In the group administered with 2-ethyl-6-methyl-3-hydroxypyridinium 2,6-dichlorophenyl(amino)phenylethanoate, on the first, fifth and eighth day after pathology simulation, the severity of neurological deficit was statistically significantly reduced by 10.2%, 12%, and 14.2%, respectively, compared to the control group (*p* < 0.05) (Figure 3).

**Figure 4:**
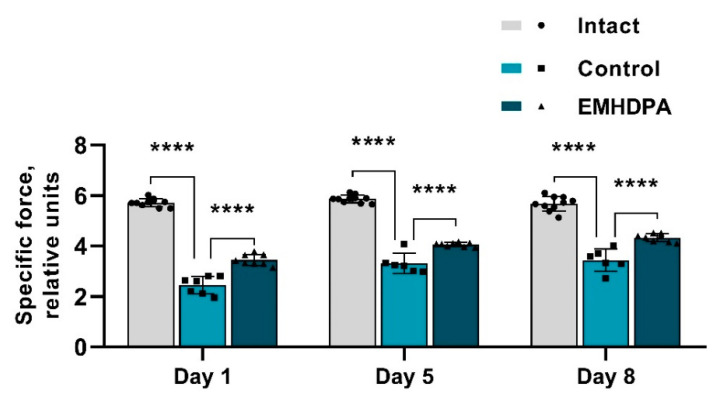
Dynamics of the indicators of specific force in the studied groups. Note: ****—*p* < 0.0001; 1—intact group; 2—control group; 3—group administered with EMHDPA 25 mg/kg.

The presented figure shows that the specific force value on the 1st, 5th and 10th day after meningitis simulation were statistically significantly higher in the group administered with EMHDPA by 17.4%, 12.8%, and 15.6%, respectively, compared to the control group (*p* < 0.05) (Figure 4).

**Figure 5:**
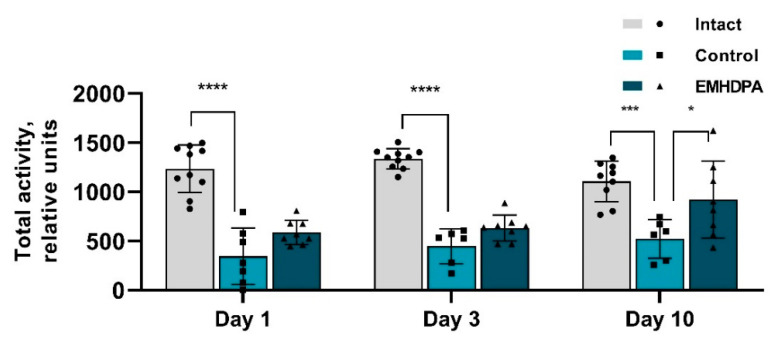
The effect of the studied drugs on the total activity in the actimetry test. Note: *—*p* < 0.05; ***—*p* < 0.001; ****—*p* < 0.0001; 1—intact group; 2—control group; 3—group administered with EMHDPA 25 mg/kg.

In the group administered with EMHDPA, total activity was higher on day 1 by 72.7%, on day 3—by 42.9%, on day 10—by 54.3% compared to the control group (*p* < 0.05) (Figure 5).

**Figure 6:**
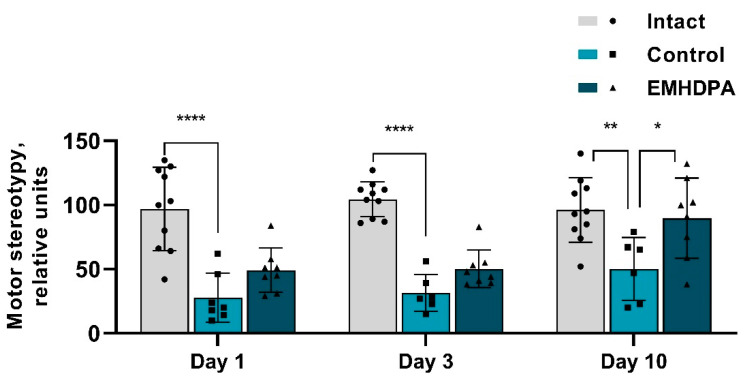
The effect of the studied drugs on the motor stereotypies in the actimetry test. Note: *—*p* < 0.05; **—*p* < 0.01; ****—*p* < 0.0001; 1—intact group; 2—control group; 3—group administered with EMHDPA 25 mg/kg.

In the group administered with EMHDPA, the number of motor stereotypies was higher on day 1st by 84.7%, on day 3rd–by 52.7%, on day 10th–by 75.2% compared to the control group (*p* < 0.05) (Figure 6).

**Figure 7:**
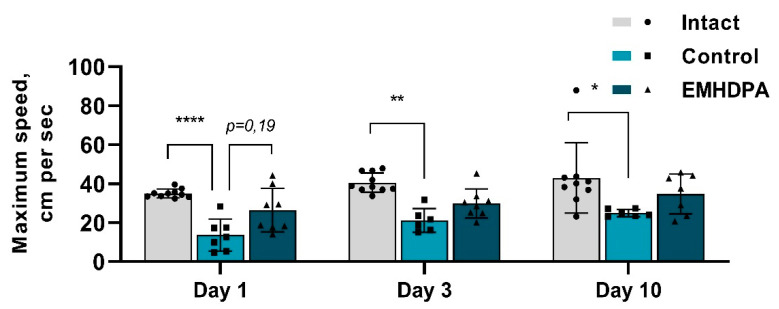
The effect of the studied drugs on the maximum speed in the actimetry test. Note: *—*p* < 0.05; **—*p* < 0.01; ****—*p* < 0.0001; 1—intact group; 2—control group; 3—group administered with EMHDPA 25 mg/kg.

In the group administered with 2-ethyl-6-methyl-3-hydroxypyridinium 2,6-dichlorophenyl(amino)phenylethanoate, the maximum speed was higher on day 1 by 104%, on day 3—by 42.6%, on day 10—by 41% compared to the control group (*p* < 0.05) (Figure 7).

**Figure 8:**
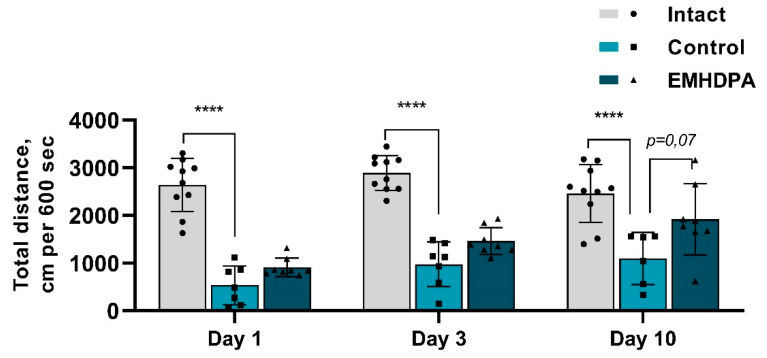
The effect of the studied drugs on the total distance in the actimetry test. Note: ****—*p* < 0.0001; 1—intact group; 2—control group; 3—group administered with EMHDPA 25 mg/kg.

According to the actimetry test, the total distance of the rats treated with EMHDPA, was higher on day 1 by 69.5%, on day 3—by 66.8%, and on day 10—by 72.2% compared to the control group (*p* < 0.05) (Figure 8).

**Figure 9:**
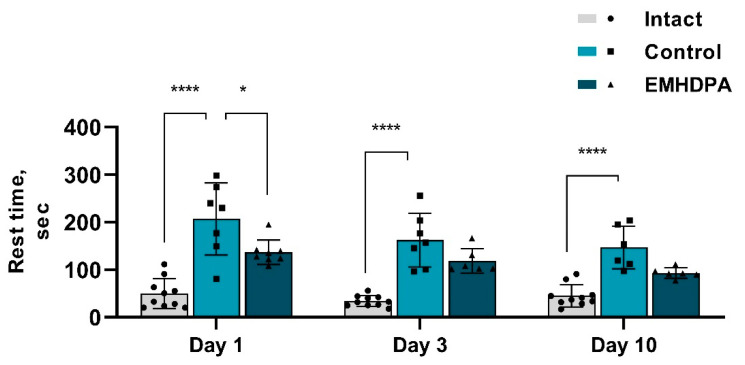
The effect of the studied drugs on the rest time in the actimetry test. Note: *—*p* < 0.05; ****—*p* < 0.0001; 1—intact group; 2—control group; 3—group administered with EMHDPA 25 mg/kg.

According to the actimetry test, in the group of rats treated with EMHDPA, the rest time was less on day 1 by 34.2%, on day 3—by 33.6%, and on day 10—by 36.7% compared to the control group (*p* < 0.05) (Figure 9).

The data described above indicate that the motor activity of animals treated with EMHDPA was significantly higher compared to the control group (*p* < 0.05). Animals in this group were more active, built up a greater speed, and went a much longer distance. The rest time in this group was shorter in comparison with the control group.

**Figure 10:**
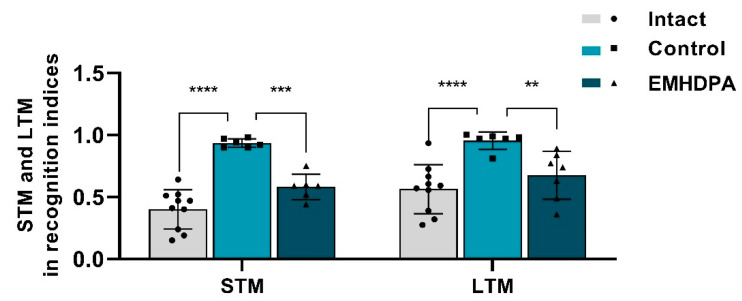
The effect of the studied drugs on the short-term memory (STM) and long-term memory (LTM) recognition indices in rats on the 10th day after meningitis simulation. Note: **—*p* < 0.01; ***—*p* < 0.001; ****—*p* < 0.0001; 1—intact group; 2—control group; 3—group administered with EMHDPA 25 mg/kg.

On the 10th day after simulated pathology, the “object recognition task” study was performed to assess the effect of drugs on the long-term (LTM) and short-term memory (STM) in rats (Figure 10). In rats treated with 2-ethyl-6-methyl-3-hydroxypyridine-2,6-dichlorophenyl(amino)phenylethanoic acid, the STM recognition index was 37.6% lower, and the LTM recognition index was 28.4% lower compared to the control group (*p* < 0.05).

**Figure 11:**
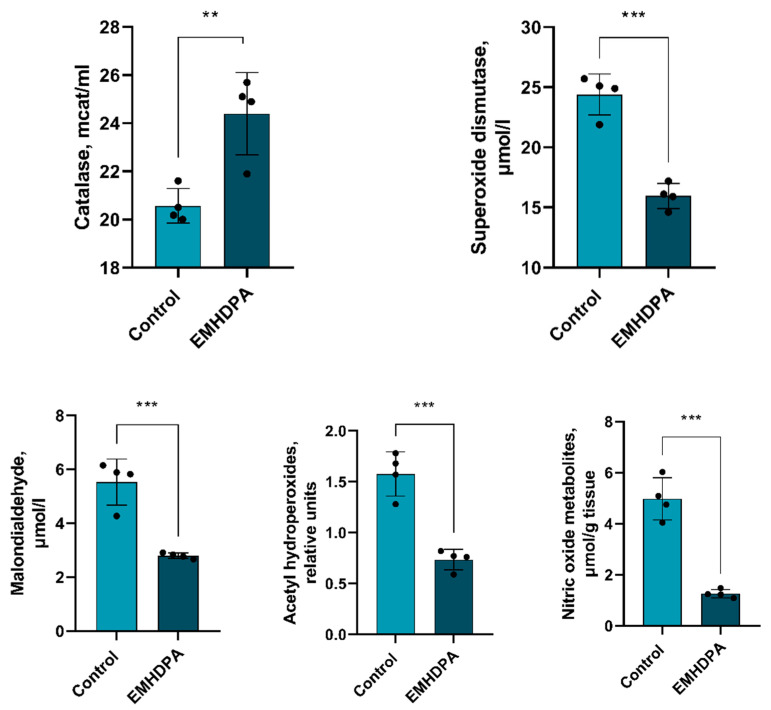
The effect of the studied drugs on the indicators of oxidative stress in the rat brain tissues. Note: **—*p* < 0.01; ***—*p* < 0.001

There is a statistically significant decrease in the activity of all estimated indicators of oxidative stress compared to the control group: a decrease in the activity of catalase—17%, superoxide dismutase—34%, malondialdehyde and acetylhydroperoxides—50%, and nitric oxide—85% (*p* < 0.05) in the group administered with EMHDPA (Figure 11).

**Figure 12:**
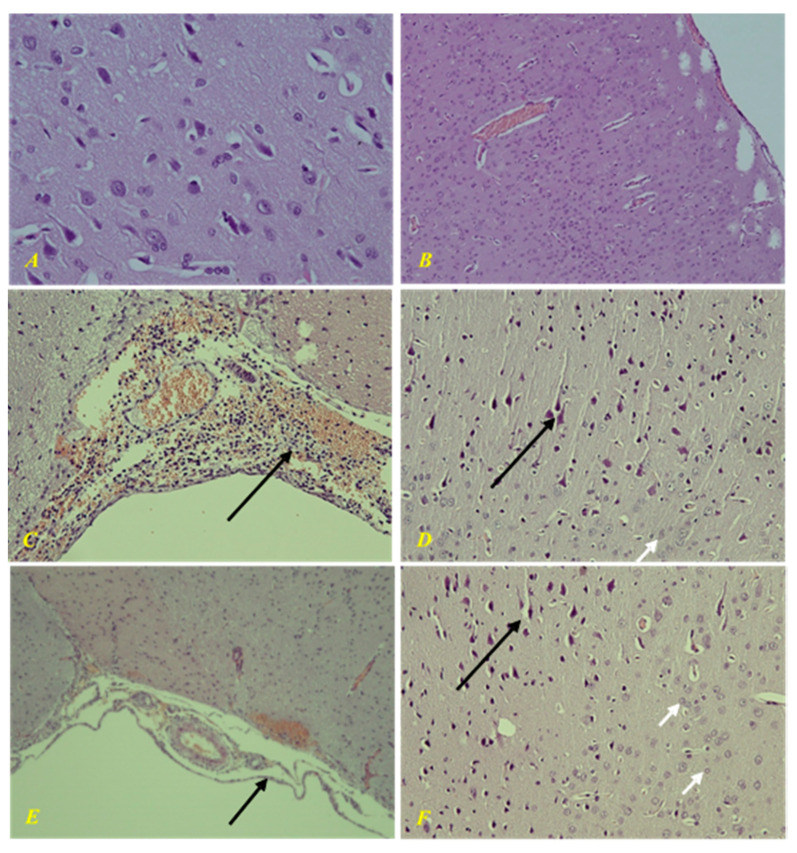
(**A**,**B**) Healthy brain, no damage; (**C**,**D**) The second group (control). (**E**,**F**) Third group (using 2-ethyl-3-methyl-3-hydroxypyridine-2,6-dichlorophenyl (amino)benzoate in a dose of 25 mg/kg). (**C**) Purulent-hemorrhagic meningitis, (**E**) Serous meningitis, black arrow—infiltration of the meninges. (**D**) Perivascular and pericellular edema, black arrow—ischemic/degenerative changes in neurons in the form of changes in the shape and size of neurons, their hyperchromia, wrinkling of individual neurons with pathological tortuosity of apical dendrites; (**F**)—with minimal changes. (**A**,**B**,**D**,**F**)—magnification: 400×, (**C**,**E**)—magnification: 200×.

In the intact group, there were no changes in the brain tissue during morphological examination (Figure 12). Morphological study of the control group showed a picture of purulent-hemorrhagic meningitis. There was severe ischemic/toxic damage to cortical neurons, perivascular and pericellular edema. In parenchymal organs, there were signs of diffuse stromal reaction to acute purulent meningitis in the form of round cell infiltration, granular, adipose, and hydropic degeneration, congestion with impaired vascular permeability.

In the group of animals treated with EMHDPA, there was a morphological picture of mild serohemorrhagic meningitis. Secondary mild ischemic/toxic damage to cortical neurons and CA1 and CA3 areas of the hippocampus, as well as moderate perivascular and pericellular edema. There were moderate dystrophic changes in parenchymal organs.

## 4. Discussion

The group administered with EMHDPA had the highest survival rate, 80%. On the first and third days after meningitis simulation, the rats treated with EMHDPA had a better clinical assessment of their health by 36.7% and 20.8%, respectively, statistically significant compared to the control group (*p* < 0.05). The restoration of clinical activity of the rats (5 points) in the group treated with EMHDPA, was the fastest on the 3rd day. The severity of neurological deficit in the group administered with EMHDPA, on the first, fifth and eighth day after pathology simulation, was statistically significantly less pronounced compared to the control group by 10.2%, 12%, and 14.2%, respectively (*p* < 0.05). The specific force values on the 1st, 5th and 10th days after meningitis simulation were significantly higher in the group treated with EMHDPA compared to the control group by 17.4%, 12.8%, and 15.6%, respectively (*p* < 0.05). In the actimetry test, the locomotor activity of animals treated with EMHDPA was significantly higher compared to the control group (*p* < 0.05). When evaluating short-term and long-term memory, it was found that rats treated with EMHDPA had lesser STM recognition index by 37.6% and LTM recognition index by 28.4% compared to the control group (*p* < 0.05).

The morphological changes were less severe in the group treated with EMHDPA.

Analysis of the data obtained during the experiment leads to the conclusion about the effectiveness of EMHDPA in the treatment of experimental PM.

In addition, EMHDPA has a positive effect on the parameters of oxidative stress in PM. In the group treated with EMHDPA, there was a decrease in the activity of oxidative stress enzymes—catalase, 17%, superoxide dismutase, 34%, malondialdehyde and acetylhydroperoxides, 50%, and nitric oxide, 85% relative to the control group (*p* < 0.05).

Thus, the obtained data confirm the cerebroprotective activity of a new pharmacological compound, EMHDPA, in the treatment of PM in the experiment. It can be assumed that this compound prevents the activation of free radicals in the development of the pathological process in the brain and blocks the lipid peroxidation of cell membranes, characteristic of PM.

Currently, two main therapeutic strategies with clinically proven effectiveness for the treatment of patients with BGM are well studied and described: optimization of antibacterial therapy and reduction of the severity of the inflammatory reaction in the subarachnoid space using adjuvant hormone therapy. In addition to rational etiotropic antibacterial therapy, the cerebroprotective component of treatment plays an important role. Studies show that oxidative stress may be associated with neurological cognitive impairment in pneumococcal meningitis [23,24].

Scientific sources cover the results of experimental animal studies of various drugs with neuroprotective properties. It is scientifically proven that the intermediate forms of reactive oxygen species and reactive nitrogen forms are formed in large quantities during the development of bacterial infection in the body [25]. Antioxidants reduce the degree of neurological damage in bacterial meningitis and are a promising strategy in the treatment of bacterial meningitis.

Currently, there is a need to closely study the existing neuroprotectors and search for new effective ones, the use of which in clinical practice would improve the course and outcome of bacterial purulent meningitis, and, consequently, improve the quality of life of patients.

Optimization of the treatment of bacterial purulent meningitis by the combined use of antibacterial drugs and neuroprotectors will reduce the duration of the acute period of the disease, reduce mortality, and reduce the degree of disability of patients. This is the direction in the treatment of BGM that we have explored in our work. EMHDPA is a new pharmacological compound with cerebroprotective properties that, when used together with an antibacterial drug in the treatment of BH, can improve the outcome of the disease. Our experimental study can serve as a basis for further clinical studies of EMHDPA.

## 5. Patents

As a part of this study, a patent of invention No. 2724883 “Method of correcting bacterial purulent meningitis by 2-ethyl-6-methyl-3-hydroxypyridinium 2,6-dichlorophenyl(amino)phenylethanoate under experimental conditions” has been obtained.

## Figures and Tables

**Table 1:** The neurological deficit assessment scale for meningitis and meningoencephalitis.

Criteria	Point			
	0	1	2	3
Spontaneous activity (in empty cage for 5 min)	No movement	Sluggish movements	Moves, but does not approach the three sides of the cage	Moves and approach the three sides of the cage
Tremor	-	Full-blown	Moderate	No
Paresis of limbs	4 limbs	2–3	1	0
Paralysis of the limbs	4 limbs	2–3	1	0
Climb the grid	-	Fail to climb	Climbs on 1/2 grids	Climbs normally
Reaction to touching the side of the body	-	No	Weak reaction	Normal reaction
Reaction to touching vibrissae	-	No	Weak reaction	Normal reaction
